# Dyslipidemia and its associated factors among adult diabetes outpatients in West Shewa zone public hospitals, Ethiopia

**DOI:** 10.1186/s12872-022-02489-w

**Published:** 2022-02-11

**Authors:** Daba Abdissa, Delessa Hirpa

**Affiliations:** 1 Department of Biomedical Sciences, College of Medical Sciences, Institute of Health Sciences, Jimma University, Oromia, Ethiopia; 2 Department of Public Health, College of Health Science and Medicine, Ambo University, Oromia, Ethiopia

**Keywords:** Dyslipidemia, Diabetes, Ethiopia

## Abstract

**Purpose:**

Dyslipidemia is a major risk factor for cardiovascular disease (CVD) in diabetic patients and early detection and treatment can reduce its morbidity and mortality. There is little information on the lipid profile of diabetic patients at West Shewa Public Hospitals, Ethiopia. Therefore, this study aimed to investigate the prevalence and related factors of dyslipidemia among adult diabetes on their follow up at West Shewa Public Hospitals, Ethiopia.

**Methods:**

A facility-based cross-sectional study was conducted from June 1 to September 30, 2020. Data were collected using pre-tested interviewer-administered structured questionnaire. The participants were recruited using a systematic random sampling method. Bivariable and multivariable binary logistic regression were employed to identify the factors associated with dependent variable. Adjusted odds ratios (AOR) were calculated at 95% confidence interval (CI) and *p* value of < 0.05 was considered as statistically significant.

**Results:**

A total number of 390 participants with a mean age of 46.45 (± 15.6) years participated in the study. The overall prevalence of dyslipidemia was 81.5% [95% CI 77.4, 85.4] and the most common lipid abnormality was elevated triglycerides (63.3%). According to multivariate analysis being female gender (AOR = 2.93; 95% CI 1.65, 5.23), age above 50 years (AOR = 3.24; 95% CI 1.54, 6.80) and alcohol consumption (AOR = 2.68; 95% CI 1.33, 5.411) were significantly associated with dyslipidemia.

**Conclusion:**

The majority of study participants had dyslipidemia. Gender, alcohol intake, and age over 50 years were significantly associated with it. Therefore, the results of this study should be taken into account in order to implement appropriate interventions for the identified risk factors.

## Introduction

Diabetes mellitus (DM) is a common metabolic disorder characterized by chronic hyperglycemia and altered metabolism of carbohydrates, fats, and proteins due to defects in insulin secretion and action, or both [1]. It is a global public health issue. In 2019, about 5 million adults died from DM and its complications [2].

Cardiovascular disease (CVD) is a major cause of morbidity and death in DM patients, and dyslipidemia is one of the major modifiable risk factors for CVD in DM patients [3, 4]. Dyslipidemia is more common in DM patients because important enzymes and lipid metabolism pathways are affected [5]. Type 2 diabetes (T2DM) increases the risk of CVD several fold. Over 50% of T2DM patients die of complications from coronary heart disease (CHD) [6].

Dyslipidemia is a collection of metabolically interrelated plasma lipid and lipoprotein abnormality involving low high-density lipoprotein cholesterol (HDL-C), high low-density lipoprotein cholesterol (LDL-C), total cholesterol (TC) and triglyceride (TG) levels. In DM patients, the most common patterns of dyslipidemia were hypertriglyceridemia, decreased HDL cholesterol levels, and elevated levels of LDL particles and it heightens the risk of CVD among DM patients [7–10]. Dyslipidemia is increasing in many developing countries due to obesity, westernization of local diets, aging populations, reduced physical activity, and other adverse lifestyle changes [11]. Lipid profile and CVD are linearly associated [12], and most (80%) of lipid disorders are associated with diet and lifestyle [13].

The cause of hypertriglyceridemia in DM patients is related to insulin resistance and hyperglycemia, which can lead to excessive production of triglyceride lipoproteins in the liver, reduced triglyceride lipoprotein clearance and in some cases, postprandial lipoprotein metabolism is impaired [14, 15]. People with dyslipidemia are twofold increased risk of CVD as compared to those with normal lipid levels [16, 17]. Besides, the causes of hypertriglyceridemia in DM patients are associated with insulin resistance and hyperglycemia, including overproduction of triglyceride lipoproteins in the liver, decreased triglyceride lipoprotein clearance, and possibly impaired postprandial lipoprotein metabolism [14, 15]. People with dyslipidemia are twice as likely to have CVD compared with normal lipid levels [16, 17].

According to previous studies the main risk factors for dyslipidemia among DM patients were hypertension, high body mass index, aging, physical inactivity and longer duration of DM [10, 18, 19]. Without timely and effective control, the rate of dyslipidemia will continue to increase, causing a heavy burden of CVD. Early detection and effective control of blood lipid levels can reduce the morbidity and mortality of CVD in patients with DM. Therefore, it is important to determine the factors associated with dyslipidemia [20]. The American Diabetes Association (ADA) recommends that all patients with DM should be evaluated for CVD risk factors at least once a year [21].

Despite the high prevalence and associated complications of dyslipidemia in DM patients, little information was available on the prevalence of dyslipidemia and related factors in diabetic patients in Ethiopia including the study area as well as they cannot differentiates between type 1 and type 2 DM. Therefore, this study aims to fill these gaps and to help clinicians and other concerned bodies to take appropriate preventive measures to reduce future complications and morbid effects on diabetic patients.

## Methods and materials

### Study design, period, area and participants

A facility-based cross-sectional study was conducted among diabetic patients attending their follow-up at chronic illness clinic of West Shewa zone public hospitals, Ethiopia from June 01 to September 30, 2020. According to projection of the 2007 census of Ethiopia, the total population of the West Shewa Zone is estimated to be 2,058,676 of which 1,028,501 are males and 1,030,175 are females in 2018/2019. In this zone, there were 520 health posts, 92 health centers, and 8 hospitals. These Hospitals were Ambo Referral Hospital, Ambo general hospitals, Gendeberet general hospital, Bako Primary hospital, Jaldu Primary hospital, Enchini Primary hospital, Gudar Primary hospital and Gedo general hospitals. Among those hospitals Ambo University Referral Hospital is the largest which provides many services for the population.

These hospitals provide internal medicine, chronic illness care laboratory, radiology, dental and pharmacy, pediatrics, family planning, maternity, gynecologic/obstetric, surgery, emergency, ambulatory clinic TB and HIV services to the people in the region. Ambo General Hospital and Ambo University Referral Hospital is located at the center of Ambo town, which is the capital of the zone and around 114 km far from the center of the country Addis Ababa to the West. All hospitals have chronic illness clinics and the number of health care workers in the area was 2967. The most common chronic diseases in the area were heart failure, DM, and hypertension.

## Sample size determination

The sample size was calculated using a single population proportion formula with 95% confidence interval, 65.6% [10] proportion and a margin of error 5% and by adding 10% for nonresponse the sample size was 382. Besides, we add eight [8] study participants were included because of they came within study period and the final sample size was 390.

### Sampling technique and procedure

There are eight public hospitals in West Shewa zone. Accordingly, Ambo University Referral Hospital was selected purposively and the other three hospitals (Ambo General Hospital, Gedo General Hospital and Guder general hospital) were selected randomly. Then, the sample was proportionally allocated for each selected hospitals (Table 1). The study subject from each selected hospitals was taken by systematic random sampling by using their medical record number as the sampling frame.

**Table 1:** Proportional allocation of sample size to randomly selected hospitals at West Shewa Zone public hospitals, Ethiopia

Selected hospitals	Number of population	Proportionally allocated samples
Ambo referral	1500	133
Ambo general	1200	107
Guder	600	53
Gedo	1000	89
Total	4300	382

### Eligibility criteria

#### Inclusion criteria

Adult DM patients (age ≥ 18 years) who had attended chronic illness clinic follow-up at the study area were included.

#### Exclusion criteria

Diabetic patients who took lipid-lowering drugs, with renal diseases, who were pregnant, newly diagnosed DM patients who were not registered, who had a known history of cardiac diseases and chronic liver diseases were excluded from the study.

### Data collection tool

Data were collected through a pretested, validated structured questionnaire which was adapted from several related literatures. It comprises behavioral factors, socio-demographic factors, anthropometric and clinical variables. Data on behavioral characteristics were collected using the World Health Organization Step wise approach for chronic disease risk factor surveillance questionnaire.

### Data collection procedures

The anthropometric measurements of each participant were performed using a standardized protocol. Participants' weight was measured in kilograms (kg) to the nearest 0.1 kg with light clothing and shoes off, and height was measured to the nearest 0.1 cm using stadiometer, and body mass index (BMI) was calculated by dividing weight in kg by the square of height. A standardized automatic electronic sphygmomanometer was used to measure the blood pressure of the participant's from the right arm. The measurement was performed 3 times and the average value was used in the analysis. Data were collected by three 3 nurses and 2 laboratory technician with supervision of 2 MPH professionals.

#### Biochemical measurement

The blood samples of 3 ml was taken from the antecubital vein under aseptic conditions using plain vacationer tubes will be obtained after an overnight fast (≥ 8 h). The blood samples will left at room temperature to allow clotting for 20–30 min and centrifuged at 3000 rpm for 10 min. The levels of glucose, TC, HDL-C, LDL-C, TG, Glycated hemoglobin (HbA1C) measured by COBAS c 311chemistry analyzer (Roche diagnostic Germany). All laboratory measurements were done as per guideline. The standardized procedures were strictly followed during the blood sample collection, storage and analysis.

### Operational definition

*Dyslipidemia* was defined as lipid profile that consists of the following abnormalities either singly or in combination. These include TC ≥ 200 mg/dL, TG levels ≥ 150 mg/dL, HDL-C < 40 mg/dL, and LDL-C ≥ 100 mg/dL.

*Hypertension* hypertension was defined as a blood pressure recording of ≥ 140/90 mmHg on more than 1 hospital visit or a documentation of treatment with anti-hypertensive medications.

*Poor glycemic control* was defined as when glycated hemoglobin level of ≥ 7%.

### Data analysis

Data were checked for errors, completeness and consistency in the hard copy, then double entered into EPI data software version 3.1 to check errors, then exported to SPSS version 22 program for analyses. Frequency, tables and descriptive summaries were used to describe the study variables. Both bivariable and multivariable logistic regression analyses were performed to identify associations between dyslipidemia and independent variables. Variables in bivariable analysis with *p* value of < 0.25 were taken as candidates for multivariable analysis. Multiple logistic regression analysis was used to identify associated risk factors for the prevalence of dyslipidemia. Crude and adjusted odds ratios and their corresponding 95%confidence intervals (CI) were computed in the bivariate and multivariable logistic regression analysis respectively. The goodness of fit of the model was checked using the Hosmer–Lemeshow test at *p* value of > 0.05 and a *p* value of < 0.05 was considered statistically significant.

### Data quality assurance

The quality of data was assured by properly designing the tool, training of data collectors and close supervision during data collection. The collected data were checked on daily basis for accuracy and completeness by principal investigator and supervisors. Two days’ training was given to the data collectors on objectives of study, ways of approaching respondents and on the data-collection tool. Pretest on 5% of the sample population was conducted at Holeta Hospital Diabetic Clinic to check consistency and applicability and after analyzing pretest results, necessary adjustments were made.

Standard operating procedure was used for all laboratory analysis of blood samples. The Internal quality control materials for each lipoproteins (HDL-C, LDL-C, TG, and TC), HbA1c were included during running each test. The tests were conducted based on the manufacturers’ instruction. The quality assurance principles for pre-analytical, analytical and post-analytical stages were applied to assure the quality result. Those intermediate results were repeatedly checked. Visual inspections of neatness of the laboratory and working bench performed to avoid cross contamination. There was properly recording of the daily result and daily follow up by principal investigator.

The results were recorded in a registration book with the individual’s bar-code in daily work. In order to avoid the errors in the results of the test, the reporting was repeatedly checked before. The quality assured results was reported to the principal investigator.

## Results

### Socio-demographic characteristics

During the study period, 390 diabetic patients participated in the study with a response rate of 100%. The mean age of the participants was 46.45 years (± 15.6). Almost half of the respondents were males (50.8%), more than three-fourth was married (76.4%) and majority of participants was urban dwellers (62.3%). Many of the respondents (51%) were Orthodox, one-third (31.5%) of them were governmental employee. Regarding participant educational status, one-third (31%) of them had primary education (Table 2).

**Table 2:** Socio-demographic characteristics of patients and prevalence of dyslipidemia participants with diabetes mellitus at West Shewa Public hospitals, West Shewa, Ethiopia

Variables	Category	Number	Percent	Outcome of dyslipidemia	
				Yes	No
Sex	Male	198	50.8	150 (75.8%)	48 (24.2%)
	Female	192	49.2	168 (87.5%)	24 (12.5%)
Age (Year)	Mean (SD)	46.45 (15.67)			
	Under 30	70	17.9	47 (67.1%)	23 (32.9%)
	30–50	171	43.8	139 (81.3%)	32 (18.7%)
	Above 50	149	38.2	132 (88.6%)	17 (11.4%)
Average monthly income (ETB)	< 1000	87	22.3	73 (83.9%)	14 (16.1%)
	2000–2999	27	6.9	22 (85.5%)	5 (18.5%)
	> 3000	193	49.5	152 (78.8%)	41 (21.2%)
Marital status	Married	298	76.4	248 (83.2%)	50 (16.8%)
	Single	73	18.7	52 (71.2%)	21 (28.8%)
	Divorced	13	3.3	92 (92.3%)	12 (7.7%)
	Widowed	6	1.5	6 (100.0%)	0 (0%)
Religion	Orthodox	199	51.0	128 (64.3%)	71 (35.7%)
	Protestant	154	39.5	100 (64.9%)	54 (35.1%)
	Muslim	22	5.6	14 (63.6%)	8 (36.4%)
	Wakefata	12	3.1	5 (41.7%)	7 (58.3%)
	Others	3	0.8	2 (66.7%)	1 (33.3%)
Educational status	Can't read and write	85	21.8	65 (76.5%)	20 (23.5%)
	Primary education (1–8)	121	31	96 (79.3%)	25 (20.7%)
	Secondary education (9–12)	70	17.9	58 (82.9%)	12 (17.1%)
	Tertiary and above	114	29.2	99 (86.8%)	15 (13.2%)
Occupational status	House wife	55	14.1	49 (89.1%)	6 (10.9%)
	Gov't employee	123	31.5	105 (85.4%)	18 (14.6%)
	private	106	27.2	81 (76.4%)	25 (23.6%)
	Farmer	106	27.2	83 (78.3%)	23 (21.7%)
Residence	Rural	147	37.7	120 (81.6%)	27 (18.4%)
	Urban	243	62.3	198 (81.5%)	45 (18.5%)
Family history of hypertension	Yes	83	21.3	66 (79.5%)	17 (20.5%)
	No	307	78.7	252 (82.1%)	55 (17.9%)
Family history of DM	Yes	105	26.9	90 (85.7%)	15 (14.3%)
	No	285	73.1	228 (80.0%)	27 (20.0%)

### Clinical and behavioral characteristics of participants

Among study participants, majority 80% of them was diagnosed with diabetes for less than 10 years. A total of 201 study participants belonged to the normal category of BMI, with 32.3% of participants being overweight. Among participants, more than half (56.9%) used a noninsulin drug and 33.1% were on insulin (Table 3).

**Table 3:** Clinical and behavioral characteristics of participants with dyslipidemia at West Shewa Public Hospitals, Ethiopia

Variables	Category	Number	Percent	Outcome of dyslipidemia	
				Yes	No
DM type	T1DM	111	28.5	81 (73.0%)	30 (27.0%)
	T2DM	279	71.5	237 (84.9%)	42 (15.1%)
Duration of DM	< 5 years	207	53.1	164 (79.2%)	43 (20.8%)
	5–10 years	107	27.4	86 (80.4%)	21 (19.6%)
	≥ 10 years	76	19.5	68 (89.5%)	8 (10.5%)
Treatment regimen	Oral hypoglycemic agents	222	56.9	187 (84.2%)	35 (15.8%)
	Injection (Insulin)	129	33.1	96 (74.4%)	33 (25.6%)
	Oral and injection	39	10.0	35 (89.7%)	4 (10.3%)
Statin treatment	Yes	37	9.5	22 (59.5%)	15 (40.5%)
	No	353	90.5	227 (64.3%)	126 (35.7%)
BMI (kg/m^2^)	Low (< 18.5)	22	5.6	20 (90.9%)	2 (9.1%)
	Normal (18.5–24.9)	201	51.5	159 (79.1%)	42 (20.9%)
	Overweight (25–29.9)	126	32.3	101 (80.2%)	25 (19.8%)
	Obese (≥ 30)	41	10.5	38 (92.7%)	3 (7.3%)
Hypertension	Yes (≥ 140/90)	170	43.3	142 (83.5%)	28 (16.5%)
	No (< 140/90)	220	56.7	176 (80.0%)	44 (20.0%)
Alcohol intake	Yes	133	34.1	119 (89.5%)	14 (10.5%)
	No	257	65.9	199 (77.4%)	58 (22.6%)
Smoking status	Yes	47	12.1	40 (85.1%)	7 (14.9%)
	No	343	87.9	278 (81.0%)	65 (19.0%)
Vigorous-intensity aerobic physical activity	Yes (≥ 75–150 min/week)	118	30.3	89 (75.4%)	29 (24.6%
	No (< 75–150 min/week)	272	69.7	229 (84.2%)	43 (15.8%)
Moderate-intensity aerobic physical activity	Yes(≥ 150–300 min/week)	141	36.2	106 (75.2%)	35 (24.8%)
	No (< 150–300 min/week)	249	63.8	112 (85.1%)	37 (14.9%)
Total cholesterol	< 200 mg/dl (Normal)	276	70.8	204 (73.9%)	72 (26.1%)
	≥ 200 mg/dl (High)	114	29.2	214 (100%)	0 (0%)
Triglyceride	Normal (< 150 mg/dl)	143	36.7	71 (49.7%)	72 (50.3%)
	High (≥ 150 mg/dl)	247	63.3	247 (100%)	0 (0%)
HDL-C	Normal (> 40 mg/dl)	203	52.1	131 (64.5%)	72 (35.5%)
	Low (≤ 40 mg/dl)	187	47.9	187 (100.0%)	0 (0%)
LDL-C	Normal (< 100 mg/dl)	325	83.3	253 (77.8%)	72 (22.2%)
	High (≥ 100 mg/dl)	65	16.7	65 (100.0%)	0 (0.0%)
Glycemic control	Good	141	36.2	122 (86.5%)	19 (13.5%)
	Poor	249	63.8	196 (78.7%)	53 (21.3%)

BMI: body mass index; LDL-C: Low density lipoprotein cholesterol; HDL-C: high-density lipoprotein cholesterol; HbA1c: Glycoslated hemoglobin; DM: diabetes mellitus

### Prevalence of dyslipidemia among diabetic patients

The overall prevalence of dyslipidemia among study participants was 81.5% [95% CI 77.4, 85.4]. Among this, the prevalence of dyslipidemia among type one and type two DM patients was found to be 73.0% and 84.9% respectively. The prevalence of dyslipidemia was higher among diabetic women 87.5% than men 75.8%. The individual lipid abnormalities were 16.7% had elevated LDL-c, 29.2% had elevated TC, and 63.3% had elevated TG and 47.9% had lowered HDL-C.

### Factors associated with dyslipidemia

On bivariable evaluation, ten variables like age, sex, physical exercise, smoking, alcohol consumption, type of DM, DM treatment regimen, Family History of DM, glycemic control and occupational status showed evidence of some association with the outcome at a *p* value of ≤ 0.25, hence included in the multivariable logistic regression analysis. The factors that were identified to be significantly associated with the dyslipidemia on multivariable logistic regression were; increased age, being female gender and alcohol consumption.

From those 1st age of respondent was one of the independent factors in predicting the dyslipidemia. Participants in their 5th decade (above 50years) were 3.24 times more likely to develop dyslipidemia compared to patients younger than 30 years (AOR = 3.24; 95% CI 1.54, 6.80) controlling for all other factors in the model. Those participants who have history of alcohol consumption were 2.68 times more likely to develop dyslipidemia than counterpart (AOR = 2.68; 95% CI 1.33, 5.41) after controlling for other variables. Finally, Female were 2.93 times more likely to develop dyslipidemia than male gender (AOR = 2.93; 95%CI 1.65, 5.23) (Table 4).

**Table 4:** Factors associated with dyslipidemia among diabetic patients at public hospitals in West Shewa, Ethiopia

Variables	Category	Dyslipidemia		Bivariable analysis			Multivariable analysis		
		Yes	No	*p* value	COR	(95% CI)	*p* value	AOR	(95% CI)
Sex	Female	168	24	0.003	0.45	0.261, 0.77	≤ 0.001*	2.93	1.65, 5.23
	Male	150	48		1	1		1	1
Age (year)	Under 30	47	23		1			1	1
	30–50	139	32	0.019	2.13	1.13, 3.90	0.089	1.77	0.92, 3.42
	Above 50	132	17	≤ 0.001	3.80	1.88, 7.70	0.002	3.24	1.54, 6.80
Occupational status	House wife	49	6	0.097	2.26	0.86, 5.90	0.091	2.42	0.87, 6.72
	Government employee	105	18	0.167	1.61	0.82, 3.19	0.386	1.38	0.67, 2.86
	Private	81	25	0.740	0.89	0.47, 1.70	0.530	0.80	0.40, 1.59
	farmer	83	23	1	1	1			1
Smoking	Yes	40	7	0.503	1.336	0.573, 3.117	0.602	1.28	0.50, 3.30
	No	278	65		1			1	1
Alcohol intake	Yes	119	14	0.005	2.477	0.1.324, 4.6	0.006	2.68	1.33, 5.41
	No	199	58		1			1	1
Type of DM	T1DM	81	30		1			1	
	T2DM	237	42	0.007	0.478	0.281, 0.815	0.890	1.09	0.29, 4.22
DM medication	Oral hypoglycemic agents	187	35		1			1	
	Injection (Insulin)	96	33	0.378	0.611	0.204, 1.826	0.727	0.64	0.38, 1.96
	Oral and injection	35	4	0.051	0.332	0.110, 1.006	0.368	1.69	0.54, 5.36
Family history of DM	Yes	90	15	0.22	1.489	0.784	0.400	1.33	0.68, 2.63
	No	228	57		1			1	
Physical activity	Yes	106	35		1			1	
	No	318	72	0.016	1.307	0.529, .887	0.063	0.59	0.34, 1.00
Glycemic control	Good	122	19		1			1	
	Poor	196	53	0.058	1.74	0.98, 3.10	0.153	1.57	0.85, 2.91

^*^Value statistically significant; AOR: adjusted odds ratio; COR-Crude odds ratio, DM: diabetes mellitus 1: reference

## Discussion

Dyslipidemia is the major independent predictor of CVD in DM patients, which results in the high mortality and morbidity of diabetic patients. It is an important modifiable risk factor for CVD and therefore, requires early screening and management as a public health priority. The purpose of our study was to determine the prevalence, pattern and predictors of dyslipidemia among diabetic patients on follow-up clinic at West Shewa public hospitals. Our study finding revealed that high prevalence of dyslipidemia among diabetic patients and with mixed dyslipidemia of TG and low HDL-C being the commonest pattern. The overall prevalence of dyslipidemia was 81.5% [95% CI 77.4, 85.4] among the study population and it was significantly associated with being female gender, alcohol intake and age above 50 years.

The overall prevalence of dyslipidemia reported in this study was comparable with a study done in Cantabria, Spain 85.3% [22] and Tanzania 83% [23]. However, it was less than the finding reported from Nepal 88.1% [19] and Thailand 88.9% [24]. The possible reasons for the variation in the prevalence of dyslipidemia might be due to differences in study population, dietary differences, sample size used and genetic differences. Similarly our study finding was higher than prevalence of dyslipidemia reported from Durame General Hospital in Southern Nations, Ethiopia 65.6% [10], China 34.64% [25], Palestine 43% [26] and in Jimma, Ethiopia 68.1% [27]. The possible reasons for those discrepancies might be due to differences in sample size, type of study population, varying dyslipidemia cut-off thresholds and sampling methods used.

In this study, dyslipidemia was significantly associated with age above 50 years. This finding was in agreement with prior studies [10, 27]. The possible reason could be ageing causes increased TC and LDL-C levels due to impaired clearance from plasma through reduced expression of hepatic LDL-C receptor [28]. Although the exact mechanism of the effect of age on blood lipid concentration is not fully understood, it may be related to the degenerative process and increased insulin resistance with age [29].

In agreement with one study [30], our study also revealed that alcohol consumption was statistically associated with the prevalence of dyslipidemia. The relationship between alcohol intake and plasma lipid levels has been studied for decades and is controversial. Excessive alcohol intake damages cholesterol homeostasis and results in increased hepatic cholesterol production and TC levels [31]. Besides one study revealed that chronic alcohol intake raises LDL-c, TG and TC levels [32]. Further studies are needed to clarify this association.

Finally, our study showed that being female gender was independently associated with dyslipidemia among diabetic patients. Although the effect of gender on dyslipidemia in patients with DM remains controversial, different studies conducted in different countries reported a higher incidence of dyslipidemia in females compared to males [10, 18, 22, 26]. Hyperlipidemia in females may be due to the effects of estrogen on body fat distribution, which results in differences in altered lipoproteins. This demonstrates the higher atherogenic risk in females than males which could be due to persistence of less favorable lipid profile [26]. Furthermore, it can be caused by excessive fat accumulation, which results from being under a considerable amount of pressure and lacking enough exercise, as men constitute the main labor force in society.

### Limitations of the study

1^st^, it was a cross-sectional survey, in which the risk factors and dyslipidemia were assessed simultaneously. Consequently, it is not possible to judge whether dyslipidemia was present before or after the proposed risk factors; Second, dietary intake was not measured, which means that the study was unable to investigate their effect on serum lipids. Thirdly, the study design allowed potential bias in the form of recall bias due to the self-reported nature of some of the information. Fourthly, the study did not take other possible risk factors (such as the use of lipid-lowering medications) into account.

## Conclusion

Our study indicated the high prevalence of dyslipidemia among diabetic patients and with mixed dyslipidemia of TG and low HDL being the commonest pattern. Female gender, alcohol intake, and age above 50 years were significantly associated with the dyslipidemia. Therefore, the results of this study should be taken into account to conduct appropriate intervention measures on identified risk factors reduction and implement routine treatments, screening and prevention of dyslipidemia.

## Data Availability

The datasets used and/or analysed during the current study available from the corresponding author on reasonable request.

## Abbreviations

ADA: American Diabetes Association
AOR: Adjusted odds ratio
BMI: Body mass index
BSC: Bachelor of Science
CHD: Coronary heart disease
CI: Confidence interval
COR: Crude odds ratio
CVD: Cardiovascular disease
DBP: Diastolic blood pressure
DM: Diabetes mellitus
ETB: Ethiopian birr
HDL-C: High-density lipoprotein cholesterol
HbA1c: Glycoslated Hemoglobin
LDL-C: Low-density lipoprotein cholesterol
mmHG: Millimeter of mercury
SBP: Systolic blood pressure
TC: Total cholesterol
TG: Triglyceride
T2DM: Type 2 diabetes mellitus
